# A Muscle-First, Electromechanical Hybrid Gait Restoration System in People With Spinal Cord Injury

**DOI:** 10.3389/frobt.2021.645588

**Published:** 2021-04-27

**Authors:** Mark Nandor, Rudi Kobetic, Musa Audu, Ron Triolo, Roger Quinn

**Affiliations:** ^1^Department of Mechanical Engineering, Case Western Reserve University, Cleveland, OH, United States; ^2^Advanced Platform Technology Center, Louis Stokes VA Medical Center, Cleveland, OH, United States; ^3^Department of Biomedical Engineering, Case Western Reserve University, Cleveland, OH, United States

**Keywords:** rehabilitation, gait, robotics, spinal cord injury, exoskeleton

## Abstract

The development of a hybrid system for people with spinal cord injuries is described. The system includes implanted neural stimulation to activate the user's otherwise paralyzed muscles, an exoskeleton with electromechanical actuators at the hips and knees, and a sensory and control system that integrates both components. We are using a muscle-first approach: The person's muscles are the primary motivator for his/her joints and the motors provide power assistance. This design philosophy led to the development of high efficiency, low friction joint actuators, and feed-forward, burst-torque control. The system was tested with two participants with spinal cord injury (SCI) and unique implanted stimulation systems. Torque burst addition was found to increase gait speed. The system was found to satisfy the main design requirements as laid out at the outset.

## 1. Introduction

It is estimated that over 17,000 new cases of spinal cord injury (SCI) occur each year in the United States alone, with an estimated 294,000 persons living in the United States with SCI (National Spinal Cord Injury Statistical Center, 2020). Restoration of standing and walking are consistently rated as top priorities for people with spinal cord injuries (SCI) (Brown-Triolo et al., 2002; Anderson, 2004; Ditunno et al., 2008). Several different technologies have been developed to address this problem, including Functional Electrical Stimulation (FES), exoskeletons, and hybrid stimulation/exoskeleton approaches. Each approach carries unique advantages and disadvantages.

A spinal cord injury is a breakdown of communication—functional electrical stimulation systems provides restoration of function via external control. Early FES systems supply current to the nerves below the level of injury to elicit muscle contractions (Kralj et al., 1980). Various systems have been developed, including some using surface electrodes (Bajd et al., 1983; Gallien et al., 1995) and others using implanted electrodes (Kobetic et al., 1997, 1999). More recent advances in the field deliver current directly to the spinal column, such as epidural spinal cord stimulation (eSCS) (Herman et al., 2002; Carhart et al., 2004) and transcutaneous spinal cord stimulation (Sayenko et al., 2014; Gerasimenko et al., 2015).

Stimulation driven muscle contractions are subject to fatigue, which can limit time spent standing and stepping (Marsolais and Edwards, 1988; Hirokawa et al., 1990). However, this approach is desirable for its physiological benefits from the users utilizing their own muscles, which maintains muscle tone and increases blood flow (Brissot et al., 2000).

An alternative solution consists of a wearable robotic exoskeleton to replace the actions of the paralyzed muscles and provide all the motive forces for locomotion. Commercialized systems include Rewalk (Esquenazi et al., 2012; Zeilig et al., 2012; Talaty et al., 2013; Benson et al., 2016; Raab et al., 2016), Ekso Bionic (Kolakowsky-Hayner, 2013; Bach Baunsgaard et al., 2018), Indego (Hartigan et al., 2015; Tefertiller et al., 2018), and HAL (Ghobrial and Wang, 2017; Jansen et al., 2018). All of these devices concentrate on replacing lost function via the exoskeleton, as opposed to restoring intrinsic function with neural stimulation. While the robotic exoskeleton solution offers predictability, reliability, and ease of control compared to FES, it does not provide the opportunity to exercise the lower limb muscles or provide the health benefits of FES.

Prior research has attempted to combine FES with multiple varieties of external bracing, with the aim of combining the strengths of bracing (consistency and reliability), with the health benefits of FES. These projects can be categorized in three varieties of increasing complexity. The first category combines FES with passive bracing, such as a reciprocating gait orthosis (RGO). The bracing is able to support the user during quiet standing without stimulation, thus delaying the onset of muscle fatigue. Because the RGO is a passive device, its joints must be manually configured. The knee joints remain locked all the time during standing and walking, and the hip joint must be manually adjusted between level gait and ramp ascent/descent (Solomonow et al., 1989).

The deficits of RGOs have been addressed by the use of semi-active braces. The joints of these devices feature electronically controlled locking/unlocking, and enough onboard sensors to automatically detect gait phases so that a control system could coordinate brace actions. Because semi-active devices rely on FES to drive all motion, they are still limited by muscle fatigue (Goldfarb et al., 2003; To et al., 2005; Chang et al., 2016).

The final category of hybrid gait restoration systems consist of an electromechanical exoskeleton combined with surface stimulation systems, such as those found in Ha et al. (2012), del Ama et al. (2014), and Alibeji et al. (2018). Unlike prior, passive work, these devices can generate power to walk above and beyond what the user's muscles can generate. With actuator redundancy, these systems present unique control challenges, but also the opportunity to incorporate greater amounts of consistency as compared to a passive or semi-passive, FES driven system. One of the challenges with these hybrid systems is understanding how to maximize the efforts of the FES system, in order to harvest the maximum physiological benefit.

Previously, we had developed a hybrid neuroprosthesis (HNP) consisting of an implanted stimulation system combined with a semi-active exoskeleton that used hydraulic cylinders and fluid to apply kinematic constraints during gait (Chang et al., 2016). It contained onboard sensors and microcontrollers to detect and control stimulation and joint constraints. It was tested with three participants with complete SCI and implanted stimulation, who were able to successfully walk from 0.03 to 0.06 m/s. The system's passive resistance and the inability of the exoskeleton to add power were noted as limiting factors. It's capacity to support high speed gait (>1 m/s) was demonstrated with able body participants, although the metabolic cost to do so was high (Chang et al., 2017).

We have attempted to combat these limitations by development of the Motor Assisted Hybrid Neuroprosthesis (MAHNP) (Reyes et al., 2020). It was conceived and designed with unique joint power units for low friction operation, high speed capability, joint locks, and retains the multi channel, implantable stimulation that is capable of generating high torque contributions from the contracting paralyzed musculature. Preliminary evaluation with two users with complete SCI was done to demonstrate system functionality, as well as show improvements to gait with the torque-pulse control.

## 2. Methods

### 2.1. MAHNP Exoskeleton Design Criteria

In order to facilitate the muscle-first control operation, it is important for actuator passive resistance to be as low as possible so that the user's muscles can backdrive the joints and move the exoskeleton. This also results in longer battery life and walking duration. We term the torque necessary to backdrive a joint as the joint's passive resistance, which is due to a combination of static friction and viscous effects. Previously, we reported on a semi-passive hydraulic exoskeleton, with passive resistance of 15 Nm at the hip joint and 6 Nm at the knee joint (Nandor et al., 2016). With that design, able bodied users were able to achieve gait speeds of 1.2 m/s. This required peak joint velocities of 142 deg/s at the hip joint, and 328 deg/s at the knee joint (Chang et al., 2017). A goal of the current work was to develop joints for the exoskeleton that improved upon the passive resistance performance of that prior design while achieving similar joint angular velocities. Additionally, because the joints were designed to have low passive resistance, they were required to include joint locks for support during stance and quiet standing. For safety reasons, the joint locks were specified to be locked while unpowered and unlocked when powered, so that a sudden loss of power would not cause the user to collapse.

#### 2.1.1. Power Transmission Components

**Figure 1:**
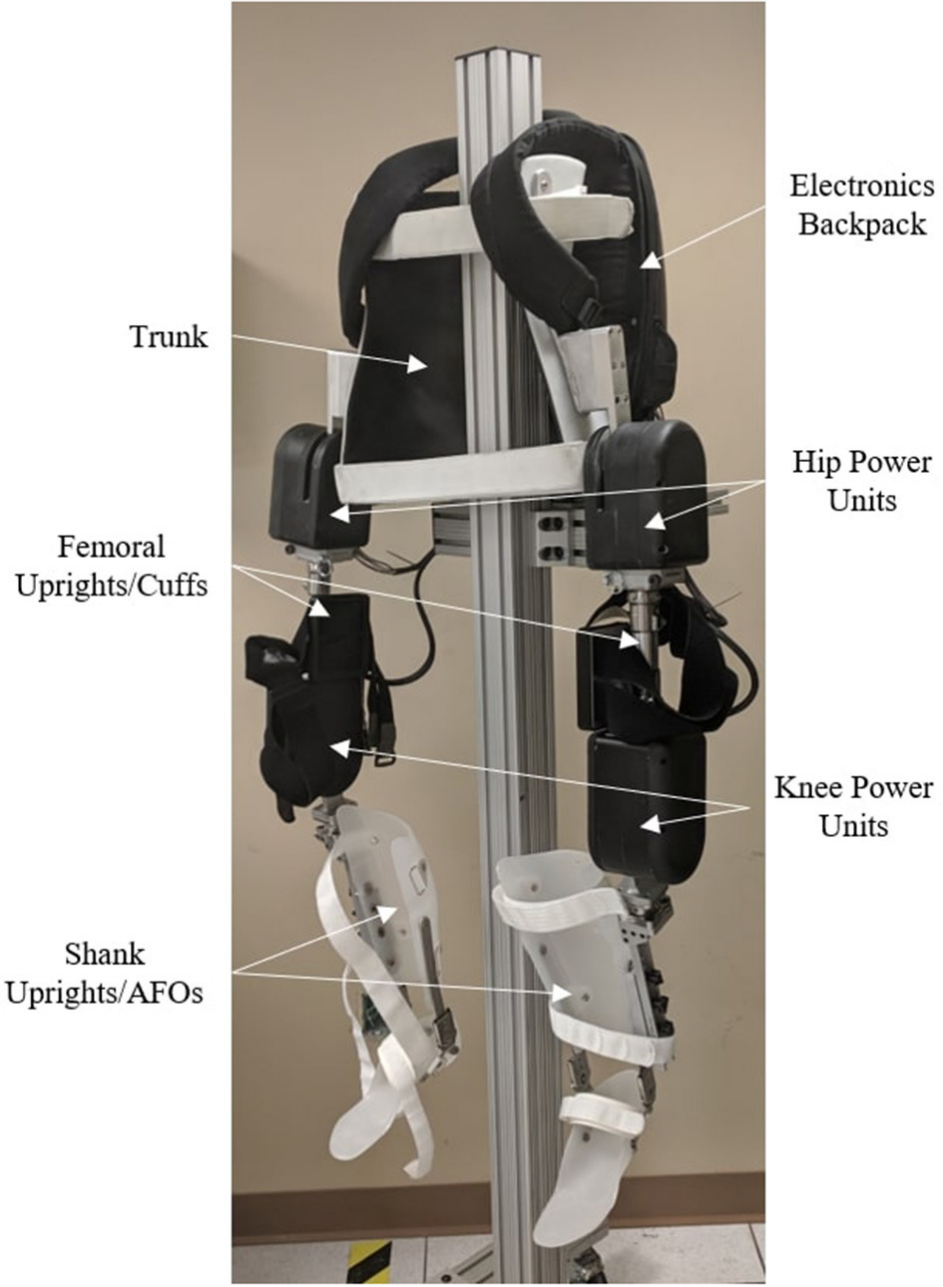
Exoskeleton for the hybrid gait restoration system.

As previously stated, the MAHNP (shown in Figure 1) consists of powered hip and knee joints. Each joint consists of a brushless DC motor, 100:1 strain wave gearing, and electromagnetic solenoid brake. At the operating battery voltage of 28.8 V, the motors have a no-load speed of 7,400 RPM, and are rated to run continuously up to 3.21 A. With the manufacturer's given torque constant, the motor is capable of 0.118 Nm of continuous torque. It is possible to supply more current (up to 15 A, limited by the controller) for brief periods of time.

**Figure 2:**
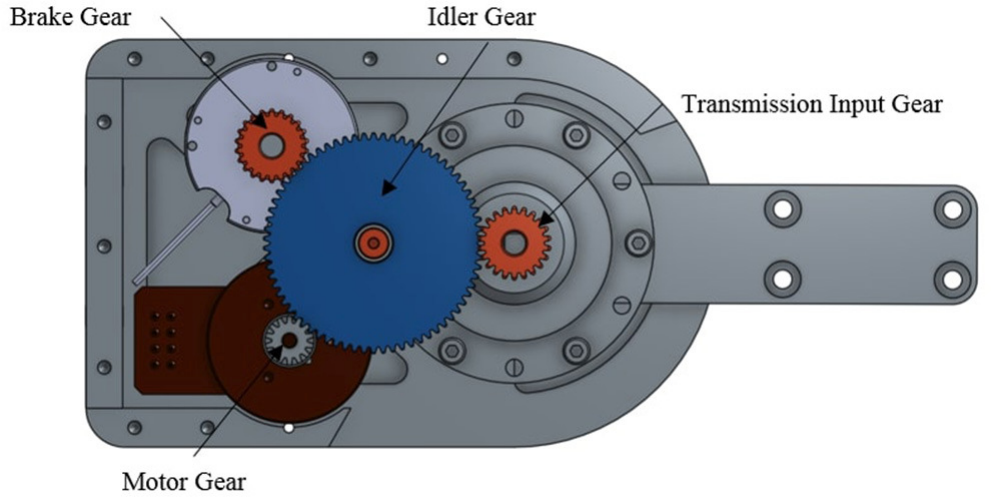
Input transmission.

The primary speed reduction transmission used in the power modules was a 100:1 speed reduction, strain-wave style transmission. Before the main harmonic drive reduction, the motor, brake, and harmonic drive input must be coupled. To transfer torque between the motor, brake, and harmonic drive input, we selected a set of spur gears, as seen in Figure 2. A large idler gear was added to rotationally link all of these components together. The higher speed requirement for the knee joints compared to hip joints allowed for a greater amount of gearing to be utilized. In the hip joint, the harmonic drive input uses a 22 tooth gear, while the motor features a 14 tooth gear. This results in overall combined gearing to 157:1. In the knee power units, the motor and harmonic drive input are coupled with identically sized gears resulting in an overall combined speed reduction (motor speed to joint speed) of 100:1.

The internal components of the power unit are contained within a two-part, machined 6061-T6 aluminum housing. These enclosures protect the user from entanglement with the gearing and sharp edges, prevent damage to internal components, and provide hard stops at extreme ranges of joint motion, which protect the user from hyper joint flexion or extension. The knee joint allows 10 degrees of extension and 120 degrees of flexion. Past experience with exoskeleton use showed that this amount of knee flexion is typically the maximum required to facilitate sit to stand transitions. The over-center action with the 10 degrees of knee extension can act as an additional brake during quiet standing, with the weight of the device driving the knee joints into the extension stops. The hip joints allow for 30 degrees of extension and 120 degrees of flexion, matching typical human range of motion. Laser sintered nylon covers protect the user from sharp edges, protect exposed components (input spur gears on the lateral side of the power unit, potentiometer on the medial side), and prevent wires from getting tangled or caught in power transmission components.

Power unit dimensional, mass, and characterization results are summarized in Table 1. The mass of each unit is approximately the same despite the gearing difference. Note that peak torque for hip and knee power units is limited by the harmonic drive to 36 Nm to prevent damage.

**Table 1:** Power unit parameter summary.

	**Hip**	**Knee**
Overall gearing (x:1)	157	100
Medial/Lateral width (mm)	105.6	103.0
Proximal/Distal width (mm)	165.2	179.4
Anterior/Posterior width (mm)	106.4	106.4
Mass (Kg)	2.2	2.2
Isometric torque (Nm/A)	4.11	2.53
Continuous torque (Nm)	13.2	8.1
Peak isometric torque	36	36
Static friction (Nm)	2.38	2.4
Viscous friction (Nm/(deg/s))	0.015	0.014

Net power unit torque is defined by the difference of the isometric power unit torque and the passive resistance torque:

(1) $$\begin{array}{l} \tau_{net}=\tau_{iso}-\tau_{fr} \end{array}$$

(2) $$\begin{array}{l} \tau_{net}=K_{\tau }I-\text{sgn}\left(\omega \right)\left(\beta \text{abs}\left(\omega \right)+f_{s}\right) \end{array}$$

**Figure 3:**
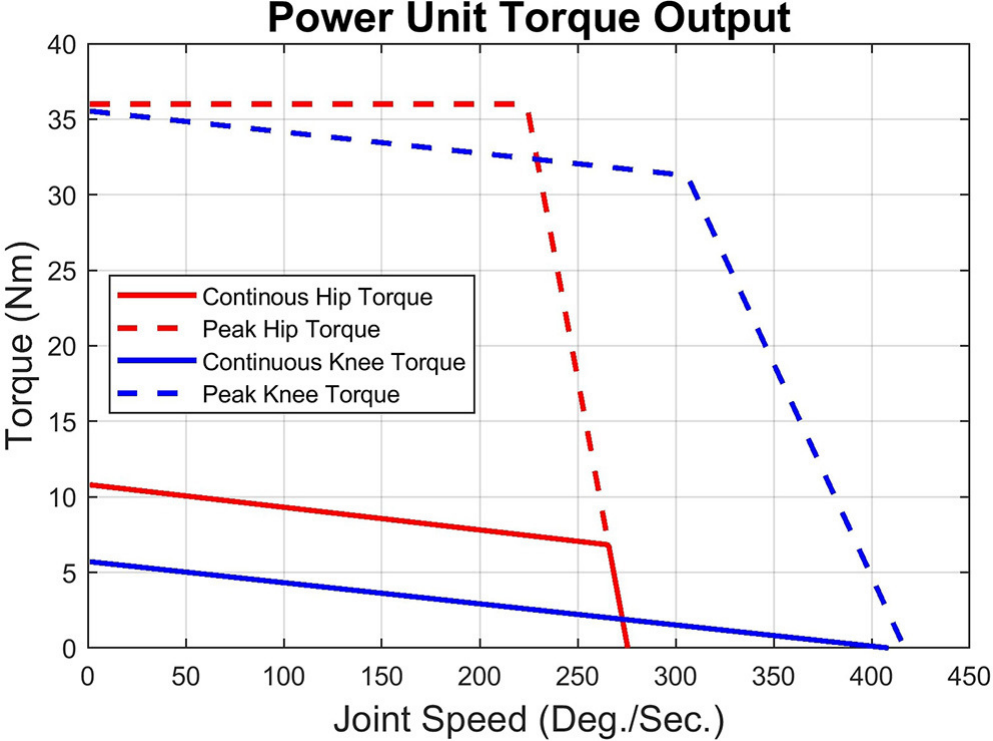
Net torque operating envelope.

Given that the motors in the power units are rated for 3.21 A continuous current, with a brief peak of 15 amps for short periods (as much as 3–5 s), this defines the continuous and peak torque and power operating envelopes for the joint power units. Equation 1 is plotted in Figure 3 to show the continuous and peak torque and power operating envelopes for the joint power units. The hip joint's peak torque is constant at 36 Nm from 0 to 220 deg/s and its continuous torque ramps down from 11 to 7 Nm at 270 deg/s. The knee joint's peak torque ramps down from 36 to 32 Nm at ~300 deg/s. Sharp declines at high joint speed occur due to system voltage saturation.

#### 2.1.2. Sensors

The sensors on the exoskeleton include the following: joint position potentiometers (part no. RH24PC E R10K L2% D5 110 mm, P3 America), motor speed sensors, motor current sensors (supplied by the motor controller), and heel/toe force sensitive resistors in the soles of the shoes.

#### 2.1.3. Electronic Components

**Figure 4:**
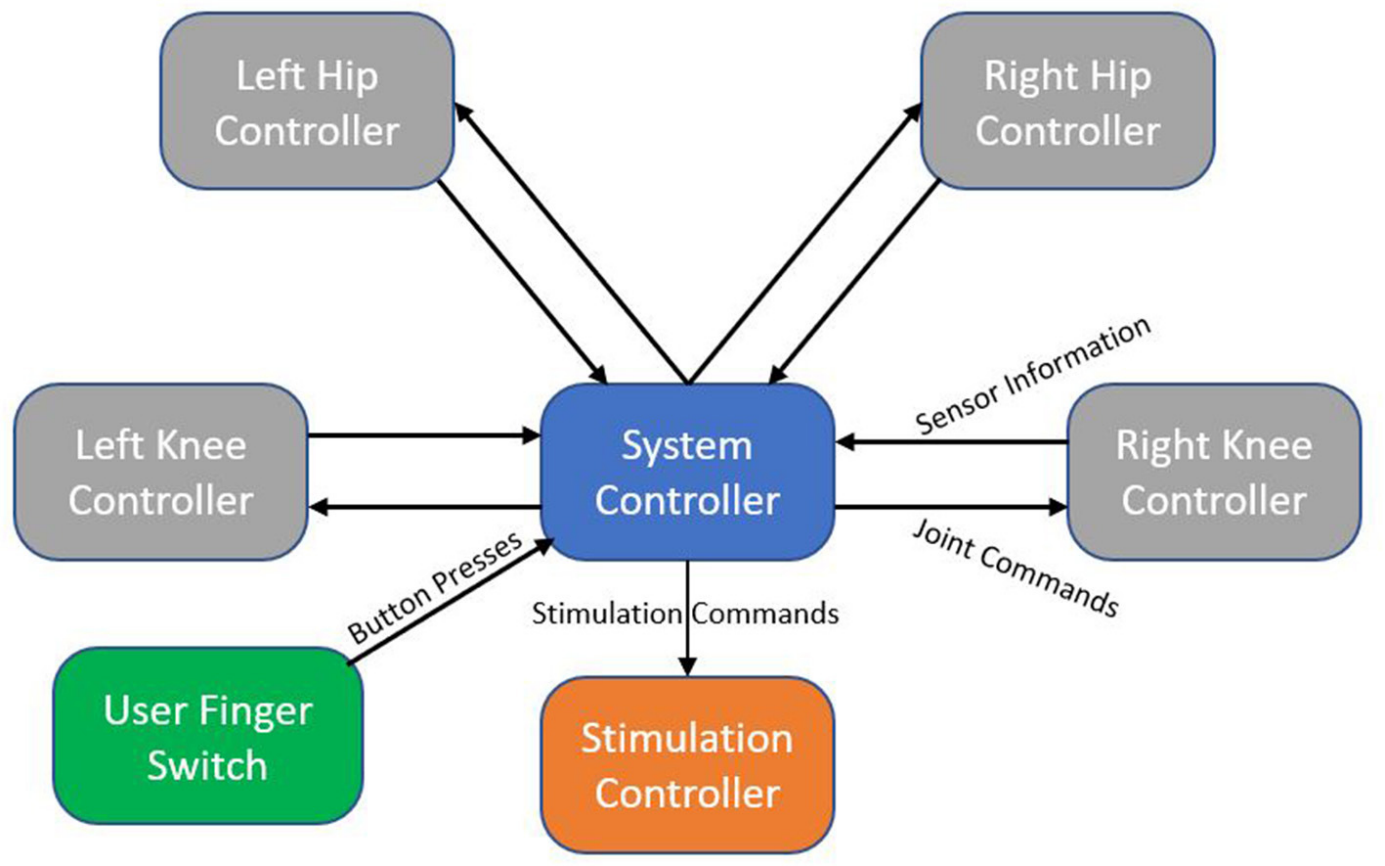
System architecture block diagram. Each node represents an independent microcontroller. Arrows show the flow of information from one node to another.

The MAHNP features distributed electronics for control, with the system architecture shown in Figure 4. Every joint contains an individual joint power module to control the motor. Each module consists of a motor controller (part no. 397172, Maxon Motors), microcontroller (Teensy 3.6, PJRC), a high power regulated 12 V power supply to control the brake, and other signal processing circuitry. The motor controller provides closed-loop current control over the motor, while the microcontroller samples the joint sensors and calculates current commands. Stimulation control is performed by its own dedicated microcontroller. High level system control is performed on its independent node. Communication between nodes is carried via system CAN bus.

A bank of two 28.8 V batteries in parallel power the entire exoskeleton (part no. PH3059HD25, Inspired Energy). These batteries are equipped with internal circuits that limit current and power output to prevent damage or injury in the event of an accidental short.

The MAHNP user carries a handheld wireless finger switch. This switch contains four different color buttons that are used to signal user intent. Communication between the finger switch and system controller is accomplished via Bluetooth.

### 2.2. System Control

System control takes advantage of the distributed architecture. Real time joint and stimulation control happen on their respective microcontrollers. Sensor information is continuously sent to the system controller, which is running a finite state machine that manages the complete system. Joint and stimulation commands are generated from the system controller and propagate down to their respective destinations.

The MAHNP joints designed to be operated in the several ways. When the joint lock is unpowered and the motor not active, the joint is locked and unable to move under external influence (this is the default setting for all joint in double stance, as well as the stance knee joint). When the joint lock is powered and the motor is not active, the joint is free to move, although the external torque applied must overcome the previously quantified passive resistance. Finally, the joints are able to operate in feed forward torque control mode, where a joint torque command is translated into a motor current command that compensates for the joint passive resistance.

#### 2.2.1. Gait Event Detection

Continuing with the theme of muscle-first operation, the MAHNP control is designed to complement the base stimulation pattern. The stimulation pattern is open loop—a pre-programmed pattern of stimulation commands as a function of time is deployed to facilitate stepping. Broadly speaking, during steps the swing leg flexors are initially active to progress the swing leg forward. In late swing, knee flexors are deactivated and knee extensors are activated to prepare the swing leg to receive weight. On the contralateral side (stance leg), hip extensors are enabled to facilitate forward progress. While in double stance, both leg knee extensors remain on to support the user until the next step is triggered. During gait with the MAHNP, this feed forward operation was kept, with exoskeleton control built on top of this. By treating the exoskeleton actions as an extension of the stimulation, this approach utilized full advantage of institutional knowledge of testing and tuning stimulation patterns and parameters for maximal effectiveness.

**Figure 5:**
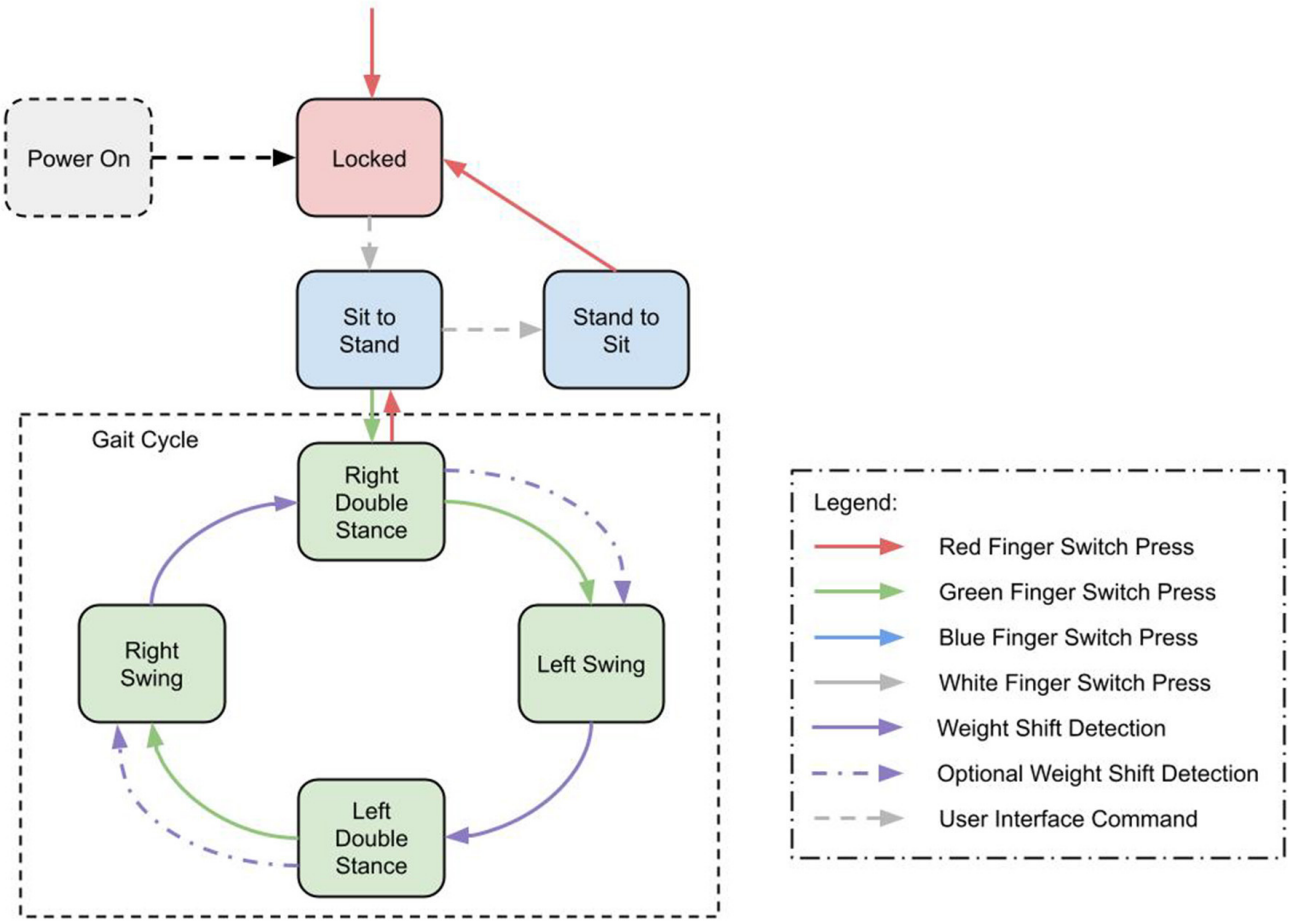
State machine diagram showing transitions between gait states and walk, stand, and sit states.

To support this we implemented a Gait Event Detector (GED) as a finite state machine to determine the phase of gait from on board sensors and trigger the correct joint power commands. The MAHNP recognized the same gait states the stimulation used; left swing, left double stance, right swing, and right double stance. These states and their one way progression can be seen in Figure 5. Progression through the state machine is achieved through a combination of user button presses via a wireless switch to initiate steps, and force sensitive resistors in the soles of the shoes to detect heelstrike. Because it is equipped with joint locks, the exoskeleton is able to support the user during double stance phases by locking its joints. The button press that begins the step stimulation pattern also triggers the exoskeleton joints to unlock. During a step, the stance knee remains locked, while the swing leg joints unlock to facilitate motion.

In addition to the gait cycle, there are transition states into and out of gait, quiet standing and sitting. All state transitions are triggered by a set of transition criteria, based on hip and knee positions and velocities, as well as the force sensitive resistors at the heels. In addition, these states can be manually commanded with a wireless tactile button interface or a smartphone interface.

### 2.3. Human Testing

**Figure 6:**
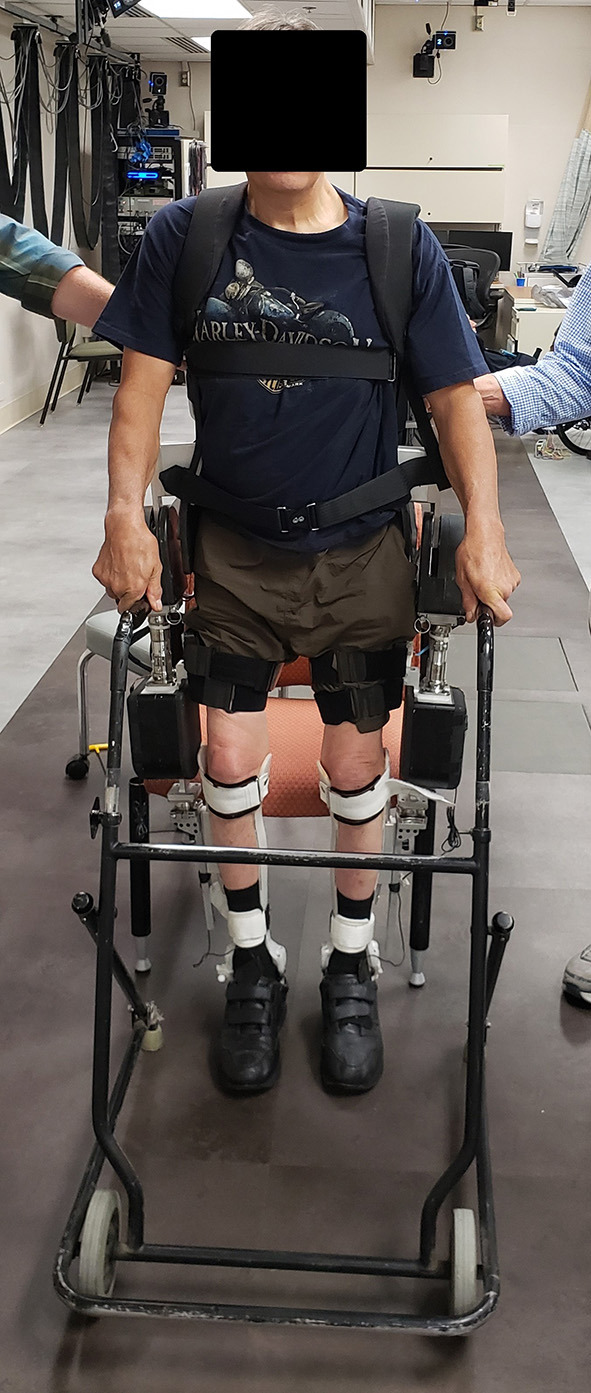
Participant with SCI standing with hybrid system.

**Figure 7:**
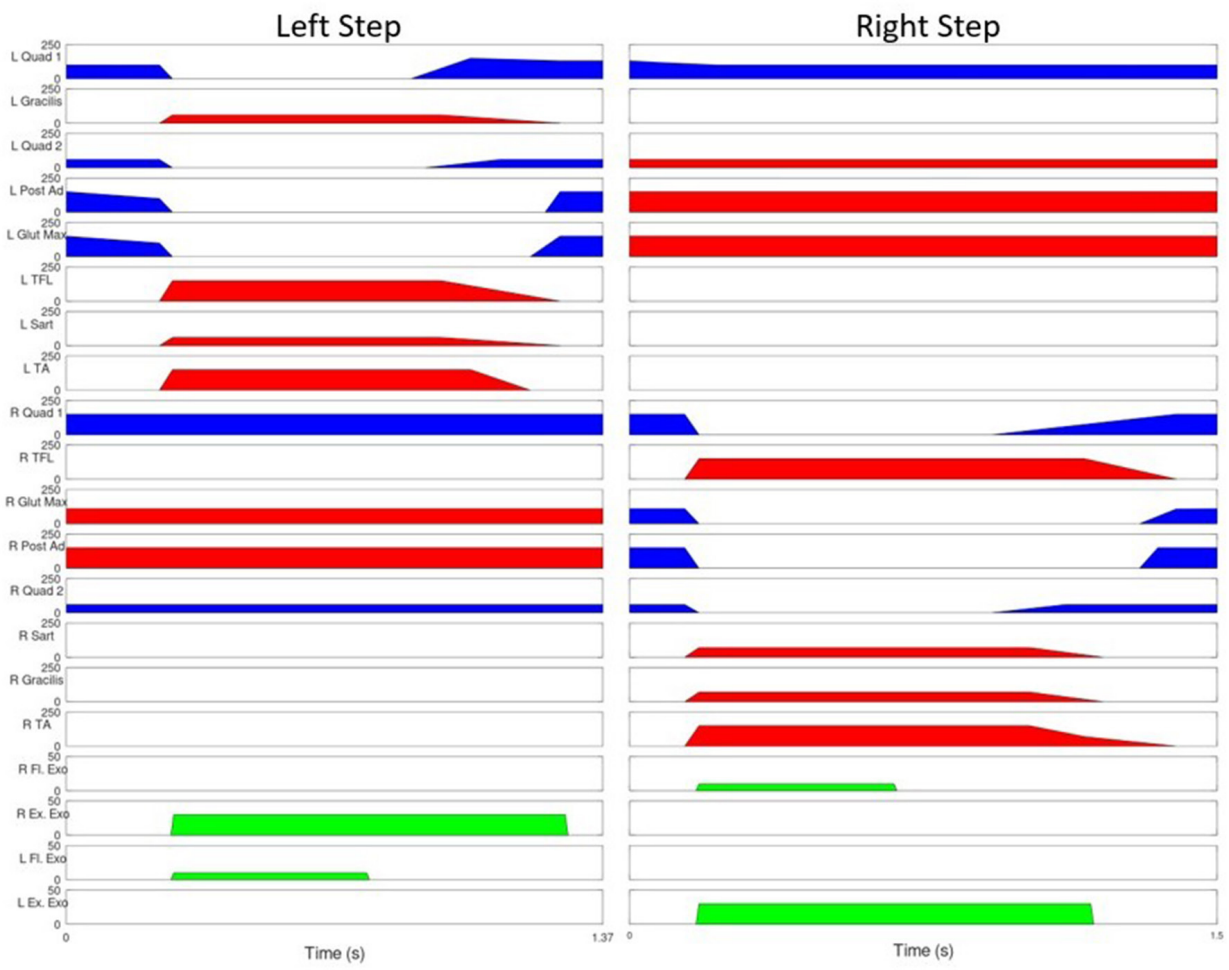
Stimulation Pattern for user with SCI. Blue represents 30 ms between pulses, red indicates 60, green indicates joint torque provided by the exoskeleton joints. The vertical lines represent the beginning of swing phase.

The MAHNP was tested with two individuals with SCI. Subject 1 had a T4 motor complete spinal cord injury (as seen in Figure 6). The subject was a 58 year old male who was 36 years post-injury at the time of the test. He had received an implanted FES system consisting of 16 chronically indwelling intramuscular electrodes Scheiner et al. (1994) that were connected to two stimulation boards on the MAHNP via percutaneous cables. These electrodes activated his hip and knee flexors and extensors and ankle dorsiflexors to complete a stepping motion (Figure 7). At the conclusion of the timed pattern, final stimulation values are held until the next step is initiated. The stimulation consisted of biphasic, charge balanced pulse trains. Pulse widths were tuned for each muscle to elicit the maximum strength while tuning out unwanted movements, with a hardware safety limit of 250 μs. Current amplitudes were set to a constant 20 mA and stimulation inter-pulse intervals of 60 and 30 ms were used depending on the muscle (Figure 7). He received the implanted system 35 years prior to the test under a separate protocol and was well-conditioned from use of the system for exercise and stepping with stimulation alone (Kobetic and Marsolais, 1994; Kobetic et al., 1997; Uhlir, et al. 2000). Prior to testing, the subject gave informed consent to participate under a protocol approved by the IRB of the Cleveland VA Medical Center. The subject is capable of gait with FES alone and a rolling walker.

Subject 2 was a 63 year old male who was 11 years post-injury. He had received an implanted FES system consisting of an implanted stimulator-telemeter (Smith et al., 1998) connected to 16 chronically indwelling intramuscular (Memberg et al., 1994) or nerve cuff (Christie et al., 2017) electrodes. Intramuscular electrodes were inserted bilaterally near motor points to stimulate gluteus maximus, hamstrings and posterior portion of adductor magnus for hip extension; gluteus medius for abduction; and sartorious and iliopsoas for hip flexion. Nerve cuff electrodes were placed around the femoral nerve for knee extension and the fibular nerve for ankle dorsiflexion. Power and command information was transmitted from the MAHNP stimulation board via a transcutaneous inductive link with a transmitting coil taped on the skin above the implant. The stimulation consisted of biphasic, charge balanced pulse trains. Pulse widths were tuned for each muscle to elicit the maximum strength while tuning out unwanted movements, with a hardware safety limit of 250 us. Current amplitudes were set to 20 mA for intramuscular electrodes and 2.1 mA for nerve cuff electrodes. He received his implant 7 years prior under a separate protocol and was well-conditioned from use of the system for exercise with stimulation alone. Prior to testing, the subject gave informed consent to participate under a protocol approved by the IRB of the Cleveland VA Medical Center. The subject is capable of a few small steps with FES alone and a rolling walker.

The MAHNP was tested under the following conditions: passive, friction compensation, and torque bursting. In passive operation, the MAHNP operated with the GED strictly locking and unlocking joints. The motors were never active. In the second operating mode (friction compensation), the passive operation was augmented with a small amount of motor operation—just enough to overcome the passive resistance of the actuators. This is achieved by sending a 0 Nm torque command to all joints. No additional torque was added to the system. The final mode of operation was a supplemental torque burst operating mode. In this configuration, the motors were ordered to provide additional assistive torque above the elimination of friction. At the beginning of each step 10 Nm burst of flexion torque was applied to both the swing hip and knee joint for 0.5 s. At the same time, the stance hip was given a 30 Nm extension torque for 1 s. Figure 7 shows Subject 1's stimulation pattern augmented with this torque burst. Torque burst values were selected via significant hand tuning across multiple trials. Both subjects participated in two test sessions. Session one consisted of exoskeleton and orthotic fitment checks, stimulation integration testing, and some preliminary walking trials across all conditions. Subjects returned for a second session dedicated to data collection. Both sessions lasted ~2 h.

A walk trial consisted of the participant standing, walking the length of the lab (10 m), and sitting down. After practice in all three control configurations, one trial of each (with the condition randomly selected) was collected for analysis. It was hypothesized that the greater levels of exoskeleton involvement would result in faster gait. We were also interested in quantifying what this involvement would cost in terms of electrical power.

## 3. Results—SCI Testing

Gait parameters, such as speed, cadence, and step length can be seen in Table 2. It shows improvement in gait speed for both subjects from the passive (baseline) condition to the most active exoskeleton (torque bursting condition. These gains come both from an increase in cadence and in step length. Elimination of passive resistance alone did not contribute to clinically significant gains in gait speed.

**Table 2:** SCI participant gait data.

	**Passive**		**Friction compensation**		**Torque bursting**	
	**Subject 1**	**Subject 2**	**Subject 1**	**Subject 2**	**Subject 1**	**Subject 2**
Gait speed (m/s)	0.11	0.11	0.11	0.15	0.41	0.20
Cadence (step/min)	18.3	18.6	20.1	25.6	45.0	29.3
Step length (m/step)	0.37	0.35	0.34	0.34	0.54	0.42
Peak knee velocity (deg/s)	131.4	82.0	120.1	105.44	247.6	215.3
Peak hip velocity (deg/s)	97.9	47.1	83.9	101.4	131.5	162.6
Hip joint excursion (deg)	41.3	14.5	37.2	20.5	51.1	64.9

*Peak velocities are calculated as the peak velocity across all steps for that condition. Joint excursion is calculated as the average maximum joint angle minus minium joint angle*.

Table 2 also shows some results of exoskeleton contribution as well. Average power was calculated as the mean joint power (motor and brake) calculated over a complete heelstrike to heelstrike step. Average joint currents were calculated similarly—mean motor currents calculated over a complete step. Note that the mean motor current for the passive trials were zero, as the joint motors were not active during this condition. Table 2 shows peak joint velocity gains from passive to torque bursting for both subjects due to added joint torque. The additional joint torque also resulted in an increase in hip joint excursion, and consequently step length. Subject 1's decrease in gait performance from passive to compensation may be attributed to fatigue, as passive was the first condition to be lasted, with compensation the last.

**Figure 8:**
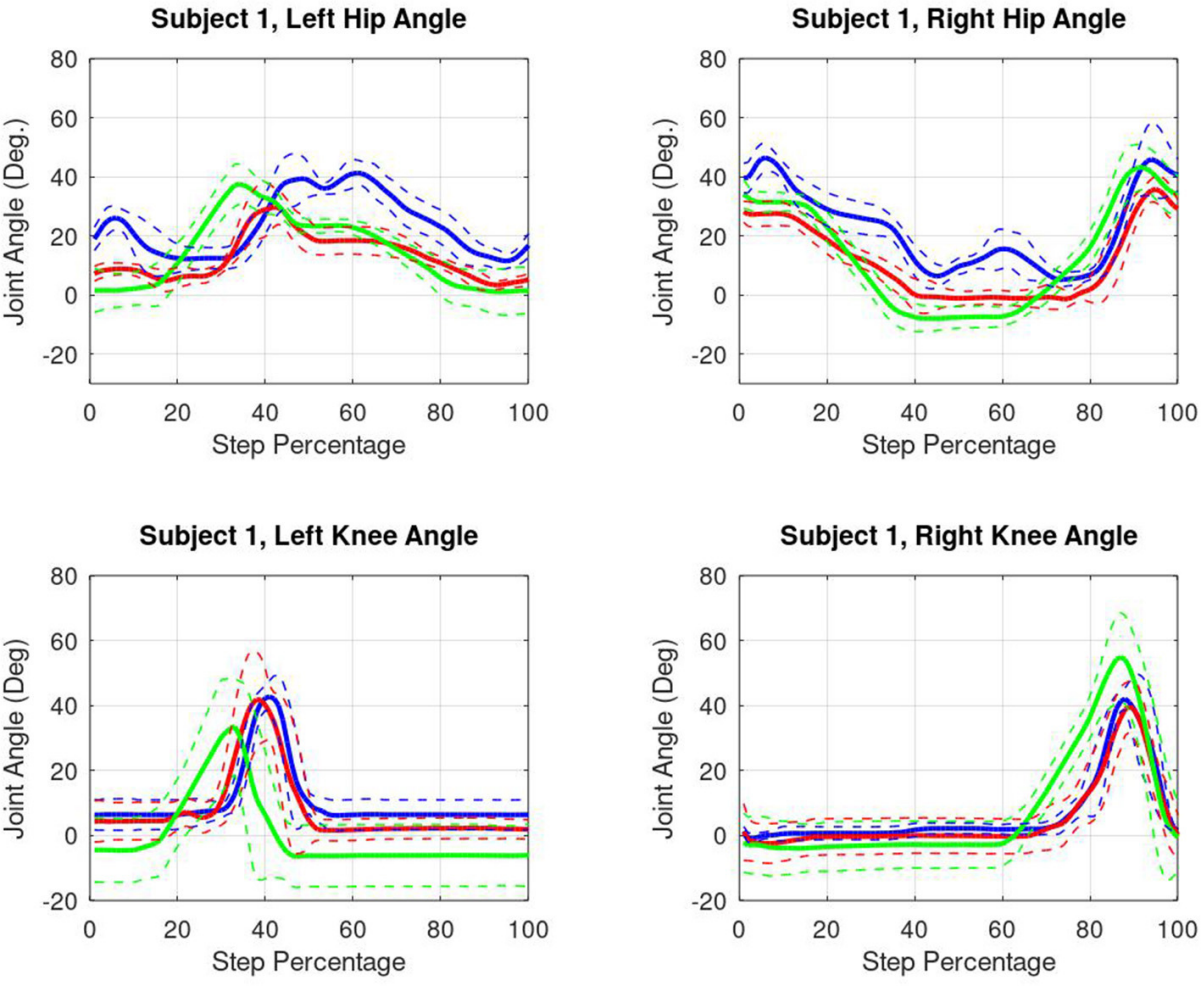
Subject 1 kinematic results. Blue lines represent passive data. Red lines represent friction compensated data. Green lines indicate torque burst data. All data is shown right heel strike to right heel strike.

Kinematic results from one subject are shown in Figure 8. All four active joints and all three conditions are shown. All data was ensemble averaged across all steps, and shown right heel strike to right heel strike, normalized across 0–100% step percentage.

Gait speed was primarily limited by control methods and the user's familiarity with the device. The user spent a considerable amount of time in double stance during each step. Whereas, able-bodied users walked continuously and dynamically, the SCI participants stepped and stopped (although this behavior was less prevalent in the torque burst condition, resulting in an increase in cadence). It is anticipated that with controller improvement and practice, gait speed can be increased.

## 4. Discussion

Gait speed did increase as the level of exoskeleton involvement increased from passive to torque burst. However, this increase does not come for free, with a significant increase in electrical power consumption. More worrying was the increase in average motor current, with a maximum value of 3.76 A average hip joint current at the highest recorded gait speed of 0.41 m/s. This exceeds the manufacturer's recommended value, and is likely to continue climbing as gait speed continues to increase. While the joints mechanically performed up to desired initial specifications, continued development will ensure this remains within safe bounds. Conversely, analysis showed that the knee joints are under utilized, with lower current demands. The opportunity here exists to potentially downsize and decrease system weight in these systems.

One review compiled gait speed results for commercially available exoskeletons. They showed that mean gait speeds for individuals with SCI was 0.28, 0.14, 0.31, and 0.16 m/s for ReWalk (Rewalk Robotics, Israel), Ekso (Ekso Bionics, USA), Indego (Parker Hannifin Corporation, USA), and the Wearable Power Assist Locomotion (Fujita Health University, Japan) devices, respectively (Louie et al., 2015). All of these devices are designed as functional replacement devices with the exoskeleton providing all the motion for gait. While there is evidence of the Indego device being used in a hybrid context with surface FES, gait speed was not reported as an outcome measure. However, the ability for the system to detect fatigue and increase exoskeleton gait contribution was demonstrated. This system was limited by using four channels of surface stimulation (Ha et al., 2016).

Systems consisting of stimulation and an unpowered exoskeleton/orthotic have been unable to match the gait speed of these stimulation only systems. One study with a mechanical controlled brake orthosis showed a maximum gait speed of 0.11 m/s (Goldfarb et al., 2003), with another utilizing hydraulic joint control mechanisms reporting 0.06 m/s maximum gait speed (Chang et al., 2016).

In this context, the MAHNP is a powered device that allows for speeds on par with what commercially available exoskeletons are capable of, with low measured joint passive resistances that still allow for the user's stimulation generated muscle torques to be the primary driver of gait. Long term usage of this device should allow users to reap the benefits of FES exercise with the exoskeleton able to supplement when muscle is fatigued.

The inclusion of subjects with implanted stimulation represents a unique and novel feature of this hybrid gait system. Each subject has 16 channels of implanted stimulation, capable of activating muscles not available to surface stimulation [a typical surface stimulation installation consists of bilateral quadriceps and hamstrings electrodes (Ha et al., 2012; Alibeji et al., 2018)]. Surface stimulation is also compatible with the MAHNP, however the exoskeleton gait torque contributions would likely need increased to compensate.

### 4.1. Future Work

It is known that ankle plantarflexion is a strong contributor to gait speed, with significant evidence to suggest a loss of plantar flexion will increase metabolic energy requirements for a given gait speed (Doets et al., 2009). It has been identified as a limiting factor in gait speed in stroke survivors (Nadeau et al., 1999). Additionally the torques required for forward propulsion due to plantarflexion are quite high, with prior research showing over 1.6 Nm/kg for able bodied subjects walking at their preferred gait speed (Winter, 1984). It is impractical to consider a large electromechanical actuator at the ankle joint (with the consequent weight and inertia penalty) that would be compatible with the stated goal of high gait speed. Rather, this is a prime opportunity for additional stimulation as an already available, zero weight/inertia penalty source of push off torque. We are exploring this possibility in future work.

This study was meant as a proof of concept, demonstrating that the proposed control system can successfully enhance the efficacy of hybrid stimulation/exoskeleton gait. The muscle-first nature of this work led to this initial study treating the exoskeleton joint actuators as an extension of the stimulation—programmed for timed bursts of high torque, similar to how stimulation is controlled. The bench testing efforts and torque controller development meant that this approach guaranteed that at no point the muscles would fight the action of the motors. While this was sufficient for initial tests, there are several limitations to this work. To date this system has only been tried on two individuals, and limited to short (10 m) walks only. Feedback was provided in a limited sense by using the onboard sensors to determine phases of gait, and adjust joint actions accordingly. Longer testing will require additional feedback in the form fatigue detection and compensation. These concepts have been demonstrated elsewhere with stimulation augmented gait in people with incomplete SCI (Müller et al., 2020). These adaptive, learning strategies for both muscle and motor contributions could be a good fit this this hybrid application as well. With these features, longer duration and distance walk trails can be completed. With a greater number of subjects completing longer walk trials, more detailed results can be presented, including user feedback, such as effort and acceptance.

## 5. Conclusion

We have designed and fabricated an exoskeleton as part of a hybrid gait restoration system that is specifically designed for high gait speed and a muscle first control strategy. Small motor, high reduction, low passive resistance actuators were developed and characterized to demonstrate their low passive resistance nature, as well as to gather data to create a feed forward torque controller. Preliminary testing of a state machine and bursting torque controller was conducted with two users with complete SCI and unique implanted stimulation systems. The addition of torque burst to the control was shows to increase gait speed compared to no torque burst conditions.

## Data Availability Statement

The raw data supporting the conclusions of this article will be made available by the authors, without undue reservation.

## Ethics Statement

The studies involving human participants were reviewed and approved by Cleveland VA Medical Center, IRB. The patients/participants provided their written informed consent to participate in this study. Written informed consent was obtained from the individual(s) for the publication of any potentially identifiable images or data included in this article.

## Author Contributions

MN contributed the engineering design work of the exoskeleton system. RK oversaw the installation and programming of the stimulation system, as well as managed the engineering development of the exoskeleton. MA, RT, and RQ advised and managed the hybrid exoskeleton program from their respective areas of expertise. All authors contributed to the article and approved the submitted version.

## Conflict of Interest

The authors declare that the research was conducted in the absence of any commercial or financial relationships that could be construed as a potential conflict of interest.
